# Comment on “Expert evaluation of GPT-4o and Gemini responses to patient questions on carotid endarterectomy”

**DOI:** 10.1590/1806-9282.20260732

**Published:** 2026-08-10

**Authors:** Mesut Engin, Ismail Sivri

**Affiliations:** 1 University of Health Sciences, Bursa Yuksek Ihtisas Training and Research Hospital, Department of Cardiovascular Surgery – Bursa, Türkiye.; 2 Kocaeli University, Umuttepe Campus, Faculty of Medicine, Department of Anatomy – Kocaeli, Türkiye.

Dear Editor,

We read with great interest the recent article by Rahman et al., In this study, the authors aimed to compare the accuracy, scientific quality, and clarity of responses generated by GPT-4o and Gemini 2.5 Flash to frequently asked patient questions regarding carotid artery disease and carotid endarterectomy. Forty unique questions were compiled from online sources and clinical experience, entered into separate sessions in Turkish, and answered in a zero-shot manner. The responses were independently and blindly evaluated by four cardiovascular surgeons using a 1–5 Likert scale across three domains: Accuracy, Scientific Quality, and Clarity. The authors further analyzed mean response lengths and the frequency of physician referral recommendations to assess the practical utility of these models in patient education. The findings suggested that both platforms provided “good” quality responses, with GPT-4o exhibiting a modest advantage in accuracy, while the overall mean scores across all evaluation domains remained statistically comparable between the two models^1^.

We would like to offer a few minor suggestions to further strengthen this research. First, the study used four different experts to rate the artificial intelligence (AI) responses. However, the paper does not show whether these experts agreed with each other. It is helpful to include an inter-rater reliability test. Using the intraclass correlation coefficient or Fleiss’ Kappa would be a great addition^2^. These tests assess whether the scores are consistent among ­reviewers. This would help confirm that the expert ratings are reliable.

Second, the authors compared the models using word counts. GPT-4o wrote longer responses than Gemini. However, a higher word count does not always mean a text is easier to understand. We suggest using a readability index for a more objective view. Formulas like the Ateşman or Bezirci-Yılmaz indices are excellent for Turkish medical texts^3^. These tools measure if a patient can truly understand the information provided by the AI.

In conclusion, while this study provides valuable insights into the potential of Large Language Models for patient education, adding objective metrics like inter-rater reliability and readability indices would further strengthen the results. We believe these additions will help researchers more effectively evaluate how AI tools can safely and effectively support ­clinical communication.
